# Changes in Tobacco Smoke Exposure following the Institution of a Smoke-Free Policy in the Boston Housing Authority

**DOI:** 10.1371/journal.pone.0137740

**Published:** 2015-09-11

**Authors:** Douglas E. Levy, Gary Adamkiewicz, Nancy A. Rigotti, Shona C. Fang, Jonathan P. Winickoff

**Affiliations:** 1 Mongan Institute for Health Policy, Massachusetts General Hospital, Boston, Massachusetts, United States of America; 2 Tobacco Research and Treatment Center, Massachusetts General Hospital, Boston, Massachusetts, United States of America; 3 Harvard Medical School, Boston, Massachusetts, United States of America; 4 Department of Environmental Health, Harvard T.H. Chan School of Public Health, Boston, Massachusetts, United States of America; 5 New England Research Institutes, Watertown, Massachusetts, United States of America; 6 Division of General Pediatrics, Massachusetts General Hospital *for* Children, Boston, Massachusetts, United States of America; Harvard Medical School, UNITED STATES

## Abstract

**Introduction:**

To protect residents from tobacco smoke exposure (TSE), the Boston Housing Authority (BHA) prohibited smoking in BHA-owned apartments beginning in 2012. Our goal was to determine if the smoke-free policy reduced TSE for non-smoking BHA residents.

**Methods:**

We compared TSE before the smoke-free policy (2012) and one year later among BHA residents as well as residents of the neighboring Cambridge Housing Authority (CHA) where no such policy was in place. Participants were a convenience sample of adult non-smoking BHA and CHA residents cohabitating with only non-smokers. Main outcomes were 7-day airborne nicotine in participants’ apartments; residents’ saliva cotinine; and residents’ self-reported TSE.

**Results:**

We enrolled 287 confirmed non-smokers (192 BHA, 95 CHA). Seventy-nine percent (229) were assessed at follow-up. At baseline, apartment and resident TSE were high in both housing authorities (detectable airborne nicotine: 46% BHA, 48% CHA; detectable saliva cotinine: 49% BHA, 70% CHA). At follow-up there were significant but similar declines in nicotine in both sites (detectable: -33% BHA, -39% CHA, p = 0.40). Detectable cotinine rose among BHA residents while declining among CHA participants (+17% BHA vs. -13% CHA, p = 0.002). Resident self-reported TSE within and outside of the housing environment decreased similarly for both BHA and CHA residents.

**Conclusions:**

Apartment air nicotine decreased after the introduction of the smoke-free policy, though the decline may not have resulted from the policy. The BHA policy did not result in reduced individual-level TSE. Unmeasured sources of non-residential TSE may have contributed to BHA residents’ cotinine levels.

## Introduction

Tobacco smoke exposure (TSE) is harmful to health, causing lung cancer, heart disease, and stroke, as well as exacerbating asthma and other respiratory illnesses.[1–2] In the United States (U.S.), approximately 37 million people live in apartments, including 2.2 million in public housing authorities serving low-income residents.[3–4] Residents of apartments (i.e., multiunit housing) are more likely to experience TSE than residents of detached housing.[5] Tobacco smoke in apartment buildings disperses throughout residences and common areas via hallways, ventilation systems, cracks and spaces in the walls and is extremely difficult if not impossible to contain using cost-effective technologies.[6–8] The issue is even more acute in public housing due to higher smoking rates in low-income groups and housing stock that may have reservoirs of tobacco smoke residue built up over years.[9–12]

With these facts in mind, in 2009, the U.S. Department of Housing and Urban Development urged public housing authorities in the U.S. to adopt smoke-free policies in all their buildings.[13] Soon after, the Boston Housing Authority (BHA) announced that it would institute a new comprehensive smoke-free policy starting in 2012 that would prohibit smoking in all residential units.[14] The goal of the policy was to reduce both non-smoking tenants’ residential TSE and resident complaints associated with tobacco smoking in BHA properties. At the time, the BHA was the largest housing authority in the country planning to institute such a policy.

To understand the impact of the BHA’s smoke-free policy on residents’ TSE, we assessed changes in objective and subjective measures of resident TSE as well as apartment air quality from before to after the BHA policy went into effect, using the neighboring Cambridge Housing Authority (CHA), which had no such smoke-free policy, as a control site.

## Methods

### Setting and Participants

The study was conducted in family housing developments within the Boston and Cambridge Housing Authorities in Massachusetts. The BHA owns 27 family developments housing approximately 18,000 residents in nearly 7,000 apartment units. The CHA, across the Charles River from Boston, owns 9 family developments housing 3,700 residents in nearly 1,500 apartment units. The family housing developments are similar in the BHA and CHA, consisting mostly of garden-style and midrise apartment buildings. Buildings in these developments are comparable in size, age, and date of most recent renovation. According to the most recent available data, approximately 19% of residents in these housing authorities are smokers, compared to approximately 14% of adults in the state of Massachusetts.[15] We recruited a convenience sample of residents from the 7 largest developments in each housing authority; each development has multiple apartment buildings. Only non-smokers living in apartment units where no residents smoked were eligible to participate in the study, as we wanted to assess the effect of the policy on the population it was designed to protect. Additional eligibility criteria were age ≥18, ability to communicate in English or Spanish, and no current use of nicotine replacement therapies. One resident per household was recruited for the study. Given the low inherent risk of participation in the study, consent was obtained verbally to facilitate recruitment. Consent was documented in the study database. The study, including the waiver of written consent, was approved by the Institutional Review Boards of Partners HealthCare and the New England Research Institutes.

### BHA Smoke-free Policy

The BHA’s smoke-free policy was implemented on September 30, 2012. According to the policy, the existing prohibition on smoking in buildings’ interior common spaces (hallways, stairways, social rooms, etc.) would be extended to include resident apartments. Smoking could also be prohibited in outdoor areas at the managers’ discretion, usually a 15 foot perimeter around buildings. The ban applies to all persons including residents, visitors, and employees. Residents in violation of the policy can be subject to fines of up to $250 and possible eviction for repeat offenses. In the year leading up to the policy change, meetings were held at every housing development to educate residents and building managers about the policy. During that period, residents were also required to sign a lease addendum acknowledging they had been informed of the policy.

### Recruitment

Participants were initially recruited by mail, with letters sent to the head of household. Additional recruitment was conducted on site at the housing authorities. The study was covered by a Certificate of Confidentiality from the National Institutes of Health to assure residents that if prohibited tobacco use was observed by or disclosed to researchers during the study, that information could not be requested by the housing authorities. After providing informed consent, residents completed an interview, provided a saliva sample, and had a passive nicotine monitor placed in the main living area of their apartments (typically the living room). Participants received $30 for completing baseline data collection. Follow-up data collection was conducted 12 months later. Participants were deemed ineligible for continued participation if they moved outside the housing authorities. Those completing the follow-up data collection received $40.

### Outcomes

Primary outcomes were measures of individual and household TSE expected to be affected by the smoke-free housing policy: self-reported TSE, saliva cotinine, and airborne nicotine in the apartment. Interviews were conducted in English or Spanish by trained interviewers using an instrument that had been pilot tested with BHA residents who were not part of the study sample. Self-reported exposure was ascertained with the following questions: “In the past 7 days, have you smelled tobacco smoke in your home? [yes/no]” and “[if yes] “In the past 7 days, on how many days have you smelled tobacco smoke in your home? [1–7]”

Cotinine is a metabolite of nicotine and is the standard biomarker for TSE.[16] Participants were asked to provide saliva samples directly into 20ml polyethylene vials. Subjects were given parafilm to chew to stimulate saliva production if necessary. Saliva samples were stored on ice in the field and transferred to -20°C freezers at the end of the fieldwork day. At the conclusion of the study, all samples were mailed to the Clinical Pharmacology Laboratory at the University of California San Francisco for high-sensitivity analysis of cotinine levels using LC-MS/MS (lower limit of detection 0.02ng/ml).[17] We deemed any individuals whose cotinine values were ≥15ng/ml at either baseline or follow-up to be current smokers and therefore ineligible for the study.[18]

Airborne nicotine was assessed using industry-standard passive monitors obtained from the University of California, Berkeley.[19–23] These monitors consist of filters treated with sodium bisulfate and enclosed in a permeable cassette through which air flows at a known rate. [24–25] After preparation, the monitors are sealed in individual containers until they are ready for deployment. Monitors were unsealed in participants’ apartments and left in place for at least 7 days after which they were collected and resealed on-site by the field staff. Monitors were then stored sealed at room temperature until the end of the study. At that point, the samples were sent to the University of California, Berkeley for analysis in which chemical constituents sorbed to the filters were extracted and analyzed by gas chromatography.[24] The passive monitor analysis had a lower limit of detection of 5ng nicotine. We estimated average apartment nicotine concentrations by assuming a constant airflow over the monitors of 24ml/minute and adjusting for the total duration of deployment. To understand factors that might affect nicotine levels, participants were asked to complete a checklist during the nicotine monitor’s deployment. The checklist ascertained the number of days windows were open, air conditioners were in operation, or tobacco was used or smelled.

The study interview also assessed non-household sources of TSE. Participants were asked about smoking that may have taken place in cars they had travelled in over the past 7 days. Participants were also asked how often in the past 7 days they smelled tobacco smoke: where they work/volunteer; in an apartment outside their building; in another apartment inside their building; in a public area inside their building; or outside the doorway of their building.

### Potential Confounders

In order to control for characteristics that differed between BHA and CHA residents, we ascertained residents’ age, sex, race, ethnicity, language, country of birth, education, occupation, marital status, number of household members (adults, children), tenure in apartment, and tenure in public housing. Race and ethnicity were self-reported into standard reporting categories for the U.S. National Institutes of Health. We also collected data on apartment characteristics including the apartment’s floor in the building and the total number of floors in the building. We used bivariate analyses to identify characteristics that differed between the BHA and CHA at the p = 0.2 level (Pearson chi-squared tests). Based on these results, we developed a model of participants’ propensity to live in the BHA in order to parsimoniously reduce bias due to confounding.[26] The propensity score model included race and ethnicity (Hispanic, non-Hispanic black, non-Hispanic white, other), country of birth (U.S. vs. foreign), marital status, employment/student status, and English or Spanish language facility as predictors. Language facility was a measure combining responses to questions asking whether the participant speaks Spanish at home or whether they speak English well/very well. Because the materials and interviews were conducted in either English or Spanish, this measure was a proxy for respondents’ relative ease with the study materials.

### Analyses

Differences in baseline characteristics were assessed using chi-squared statistics. Unadjusted analyses of main outcomes and non-household sources of TSE are presented as within-housing authority averages for each time point. To assess the effect of the BHA’s smoke-free policy on residential TSE, we modeled differences in within-person/residence changes in outcomes with regression models that included subject-level random effects. Based on the Surgeon General’s assessment that there is no safe level of TSE, we dichotomized each of our outcomes into “detectable” vs. “not detectable” levels of exposure. Detectable exposure was modeled using logistic regression. We also modeled each outcome as a continuous measure. Following standard methods for left-censored data,[5, 11, 27] we modeled log-cotinine and log-nicotine using interval regression (a generalization of tobit regression). The number of days in the past 7 that the respondent reported smelling smoke in the apartment was modeled using linear regression. Because we used random effects models, all observed outcomes were included in the models, even when participants were observed only at baseline. All models included indicators for housing authority (BHA vs. CHA), policy period (pre vs. post), and an interaction term for housing authority*policy period. An interaction term significantly different from 0 at the 0.05 level was considered evidence that changes from before to after the introduction of the BHA smoke-free policy were different across housing authorities. All models controlled for subjects’ quintile of the propensity score. Models for cotinine and number of days smelling smoke further controlled for education, tenure in the apartment, and the floors in the building. Models for nicotine were adjusted for whether windows were open during sampling and the resident’s tenure in the apartment. We conducted sensitivity analyses to determine whether our findings changed when analyzing only those with complete data on all outcomes versus those with complete data on particular outcomes. All analyses were conducted in 2014 using SAS 9.3 (Cary, NC) except for the interval regression analyses which were performed using Stata 12.1 (College Station, TX).

## Results

In the summer and early fall of 2012, we consented 201 participants from the BHA and 98 from the CHA. Two subjects from the BHA dropped out at baseline because they failed to complete the data collection. Ten participants (7 BHA, 3 CHA) were determined to be ineligible for the study after enrollment when cotinine analysis suggested active smoking (cotinine>15ng/mL). After these exclusions, there were 192 participants from the BHA and 95 participants from the CHA. Residents of the BHA and CHA were different on a number of key characteristics at baseline (Table 1). Participants from the BHA were more likely to be women, of Hispanic/Latino ethnicity and to speak Spanish, less likely to be black/African-American, had lower education, had greater facility with English and/or Spanish, and were less likely to be full-times students/employees. After propensity score adjustment, none of these characteristics was significantly different between the BHA and CHA residents. Of the subjects meeting eligibility criteria and providing baseline data, 7 moved out of the housing authorities during the study year, and 51 were lost to follow-up for a retention rate of 79%. Those lost to follow-up were similar to those continuing participation except they were slightly more likely to be black/African-American and less likely to be of “other” race (p = 0.04).

**Table 1:** Participant characteristics.

*Characteristics*		*Cambridge (n = 95)*	*Boston (n = 192)*	*P value*	*P-value after propensity score adjustment*
Age in years; mean±sd		49.0 ± 15.7	49.9 ± 15.4	0.64	0.35
Gender; n (%)				0.005	0.14
	Female	69 (72.6)	167 (87.0)		
Hispanic; n (%)		18 (19.1)	131 (68.2)	<0.001	0.94
Race; n (%)				<0.001	0.96
	White	12 (12.8)	27 (14.4)		
	Black	57 (60.6)	45 (24.1)		
	Other	23 (24.5)	107 (57.2)		
	More than one	2 (2.1)	8 (4.3)		
English or Spanish language facility; n (%)		77 (81.1)	190 (99.0)	<0.001	0.12
Employed or full time student; n (%)		54 (56.8)	69 (35.9)	<0.001	0.72
Marital status; n (%)				0.08	0.96
	Married or living with a partner	39 (41.1)	53 (27.6)		
Education; n (%)				0.02	0.29
	≤ GED or equivalent high school graduate	55 (58.5)	134 (70.9)		
	Some college, no degree or associate degree	26 (27.7)	46 (24.3)		
	≥ Bachelor’s degree	13 (13.8)	9 (4.8)		
Born in the US; n (%)		29 (30.5)	66 (34.4)	0.59	0.50
Live in the apartment for ≥ 10 years; n (%)		22 (23.2)	67 (34.9)	0.06	0.44
Floor of building; median (IQR)		2 (1,3)	2 (1,4)	0.13	0.20

**Fig 1:**
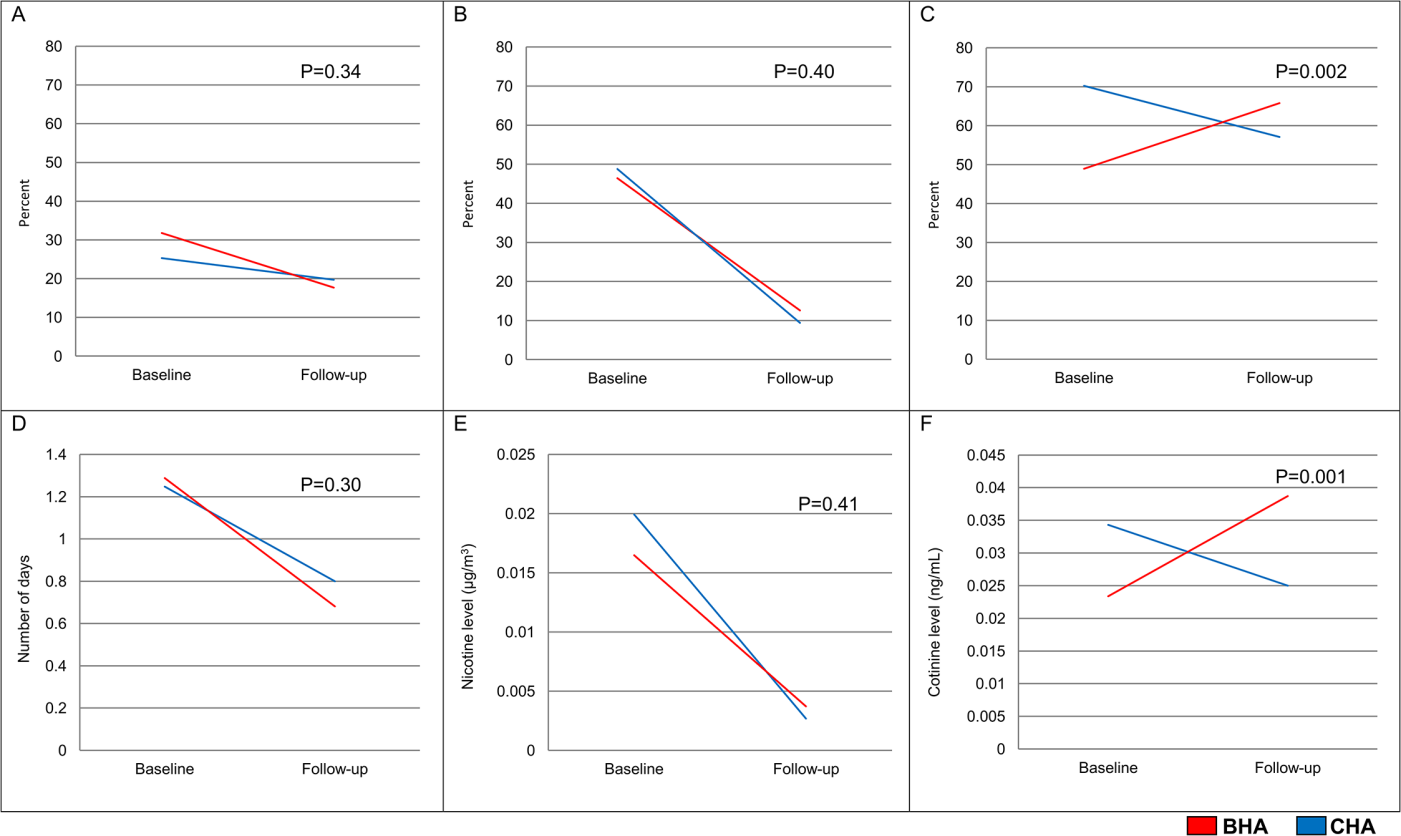
Changes in Subjective and Objective TSE from Baseline to Follow-up^a^. (A) % Residents who smell smoke in their apartments. (B) Apartment Nicotine (% Detectable). (C) Residents’ Cotinine (% Detectable). (D) Self-report (days). (E) Apartments’ Nicotine (μg/m^3^). (F) Residents’ Cotinine (ng/mL). ^a^Graphs are unadjusted, P-values are regression-adjusted.

At baseline, a slightly higher proportion of BHA (32%) than CHA (25%) residents reported having smelled tobacco smoke in their apartments over the prior 7 days (Fig 1A). Over the course of the study year, this level fell by 14 percentage points in the BHA, while in the CHA, it fell by 6 percentage points. These changes were not significantly different from one another in our regression-adjusted analyses (p = 0.34). The proportion of households with detectable airborne nicotine was high in both housing authorities at baseline (46% BHA and 48% CHA) (Fig 1B). By the follow-up period, detectable nicotine had decreased substantially at both housing authorities to 13% in the BHA and 9% in the CHA. Again, these changes were not statistically different from one another in adjusted analyses (p = 0.40). The proportion of residents with detectable cotinine at baseline was also high in both housing authorities, but was lower among BHA residents than CHA residents (49% vs. 70%, p = 0.08) (Fig 1C). When measured at follow-up, the proportion of BHA residents with detectable cotinine had risen by 17 percentage points while the proportion of CHA residents declined by 13 percentage points. This difference was significant in the adjusted analysis (p = 0.002). Panels d-f of Fig 1 show similar trends in mean (days smelled smoke in past 7) and geometric mean (μg/m^3^ nicotine, ng/mL cotinine) outcomes. Changes in nicotine and changes in cotinine were not correlated (Pearson’s r = 0.09, p = 0.15).

**Fig 2:**
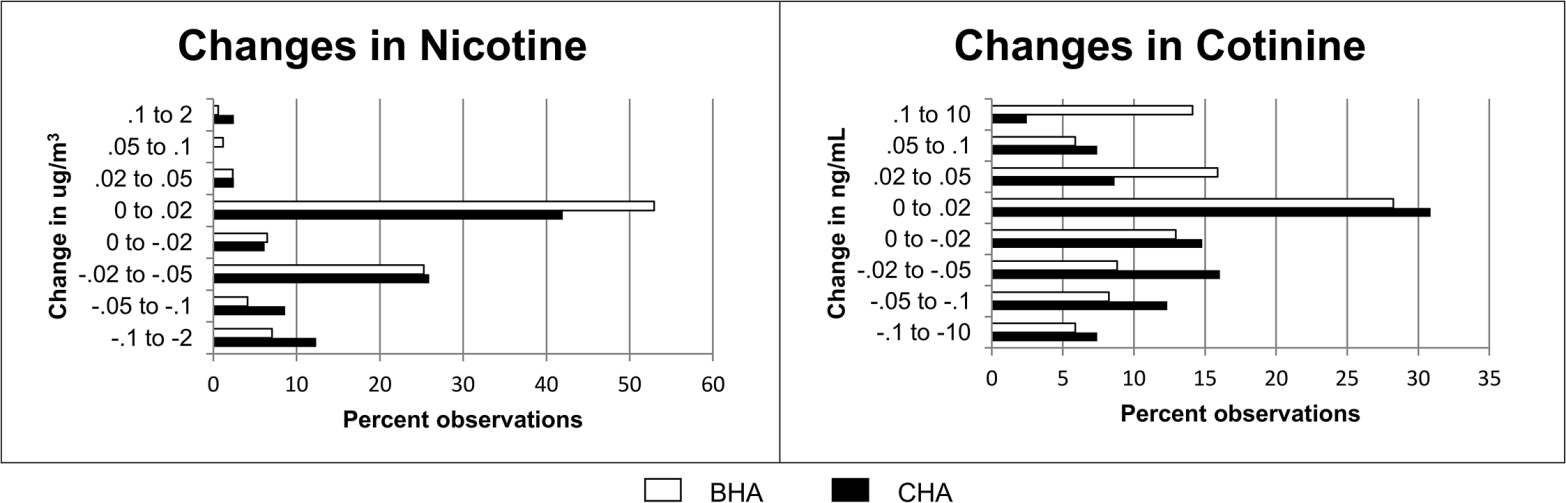
Distribution of changes in outcomes from Baseline to Follow-up.

Fig 2 illustrates the distribution of changes in continuous outcome values from baseline to follow-up for each of our objective outcomes assuming values below the limit of detection are 0. The change values are grouped in a quasi-log-transformed manner to compress the distribution. The great majority of changes were between -0.05ng/mL and 0.05ng/mL for cotinine and between -0.05μg/m^3^ and 0.05μg/m^3^ for nicotine. Neither distribution had large densities at the extremes.

**Fig 3:**
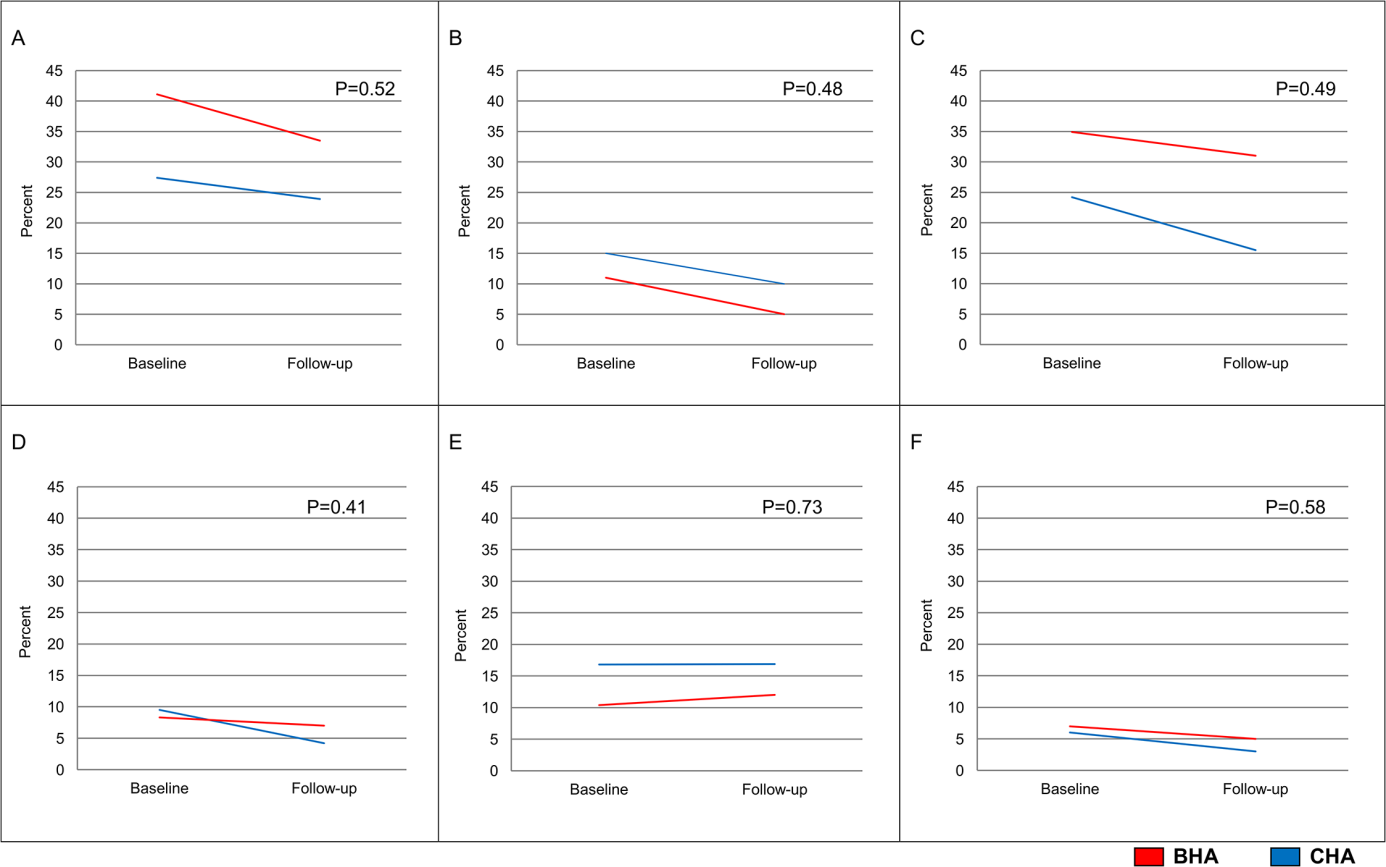
Changes in Self-reported TSE at Non-household Sites^b^. (A) % Residents smell smoke outside doorways of their buildings *often or all of the time* (in past 7 days). (B) % Residents smell smoke at place they work or volunteer *often or all of the time* (in past 7 days). (C) % Residents smell smoke in public areas of their buildings *often or all of the time* (in past 7 days). (D) % Residents smell smoke at peoples’ home inside the building *often or all of the time* (in past 7 days). (E) % Residents smell smoke at peoples’ home outside the building *often or all of the time* (in past 7 days). (F) % Residents riding in car with smoking present (in past 7 days). ^b^Graphs are unadjusted, P-values are regression-adjusted.

Fig 3 illustrates changes in potential non-household sources of residents’ TSE. Most changes were qualitatively similar for each housing authority. In sensitivity analyses, we included each of these potential sources of TSE as well as apartment nicotine as independent variables in the regression models estimating changes in cotinine. Adjusted differences in cotinine did not change quantitatively or qualitatively with the inclusion of these factors, suggesting none was a mediator of the relationship between living in the BHA and changes in cotinine following the introduction of the smoke-free policy (results not shown).

## Discussion

In 2012, the Boston Housing Authority became the largest public housing authority in the U.S. to prohibit smoking within residential units. To our knowledge, this is the first study to assess changes in a broad selection of objective and subjective measures of TSE among non-smokers following the introduction of a smoke-free housing policy.

The concentration of airborne nicotine in apartments is the objective study outcome most directly related to the smoke-free policy. We found substantial reductions in airborne nicotine within BHA households from the period immediately prior to the policy implementation to approximately a year later. This longitudinal change is consistent with published studies of cross-sectional differences between smoking-allowed and smoke-free buildings in the BHA and other public housing authorities.[7–8, 28] However, we observed similar reductions within the CHA as well; we are thus unable to attribute the change in Boston entirely to the smoke-free policy. It is unclear what may have driven nicotine levels down in the CHA. During our follow-up data collection, the CHA began a dialogue with its residents about a future smoke-free policy for the authority. It is possible that smokers in the CHA reduced smoking inside CHA buildings in anticipation of the policy (which was not implemented until the summer of 2014).There were no other city- or housing authority-wide initiatives in Cambridge that would have been expected to affect apartment nicotine levels.

Mean TSE for residents at each site and time period, as measured by salivary cotinine, was consistent with the low values observed in the general US population, contrary to preliminary studies which found higher levels of TSE in public housing than elsewhere.[27, 29] We observed a rise in BHA residents’ cotinine while cotinine declined among CHA residents. A concern prior to implementation was that the BHA’s smoke-free policy might have unintended consequences if it forced smoking activity out of smokers’ homes and into spaces used by non-smokers and smokers alike, such as around building doorways or other common areas. Our secondary analyses provide no evidence to support this concern. There were no significant changes in residents’ reports of smelling smoke around doorways or other areas around their buildings that would explain the differential changes in cotinine levels for BHA versus CHA residents. However, a relatively small increase in the number of residents smoking around building entrances and walkways could have increased TSE without residents necessarily noticing. Self-reported assessments of TSE off of housing authority property, such as on surrounding sidewalks, in cars, in friends’ homes, or at places of employment or volunteering did not explain the differential changes in cotinine levels across sites.

For a residential smoke-free policy in public housing to reduce residents’ overall TSE, there must be compliance with the policy and residential TSE must make up a significant proportion of residents’ overall TSE. Enforcement of smoke-free policies is challenging because there are relatively few personnel and they are responsible for enforcing the policy over properties covering large areas. In addition, the standard of proof for documenting violations may be high because public housing is a resource of last resort for many residents and housing authorities are reluctant to render residents potentially homeless. The disciplinary processes for adjudicating alleged policy violations can be time-consuming. Nevertheless, the observed decrease in air nicotine among BHA apartments suggests that lack of compliance was not a major issue.

Whether residential TSE makes up a high proportion of overall TSE for public housing residents is unclear from our data. The reduction in apartment nicotine in the BHA over the study period coupled with the increase in saliva cotinine there indicates that exposure was taking place outside of residents’ apartments. While our self-reported measures of TSE suggest there was no increase in TSE taking place on housing authority property, it is possible that these measures were not sensitive or specific enough to adequately capture TSE elsewhere on BHA property. It is also possible that the observed increase in saliva cotinine among study participants living in the BHA are due to exposures that took place off of BHA property. The true effect of the BHA policy on residential TSE may emerge over time. However, our results suggest that simply having a smoke-free policy in all indoor locations will not guarantee significant short term changes to overall resident TSE even when the measured nicotine level in units decreases. As the largest public housing authority in the country at the time to implement a smoke-free policy, the BHA had little precedent to guide it and has had to make ongoing adjustments to its policy implementation.

Strengths of our study include assessment of a compulsory smoke-free policy (mitigating selection effects within the BHA) and concurrent collection of multiple objective and self-reported measures of TSE. Nevertheless, because of its observational design, we cannot rule out confounding due to differences between the BHA and CHA populations or their circumstances. For example, approximately 10% of residents move out of public housing each year; we do not know whether smokers disproportionately moved out of or non-smokers disproportionately moved into the BHA. We made an effort to protect against observed confounders by using propensity score methods and by examining within household/resident changes over time. In addition, by comparing housing authorities in neighboring cities we attempted to mitigate or eliminate differences in environmental factors that could affect TSE (e.g., physical environment, state policies, or large area secular trends).

In conclusion, there was a substantial reduction in air nicotine in BHA apartments associated with the introduction of the smoke-free policy. However, CHA apartments, which had an impending but not yet implemented smoke-free policy during the study period, also had a contemporaneous decline in nicotine. Therefore, the decline in air nicotine in the BHA apartments cannot be unequivocally attributed to the smoke-free policy. Furthermore, the BHA policy did not result in reduced individual-level TSE. Future research is needed to examine the effect of smoke-free policies on other constituencies of public housing such as current smokers, children, and those living in housing designated for elderly and disabled residents.

## References

[1] U.S. Department of Health and Human Services. The Health Consequences of Involuntary Exposure to Tobacco Smoke: A Report of the Surgeon General,. In: Public Health Service CfDCaP, National Center for Chronic Disease Prevention and Health Promotion, Office on Smoking and Health, editor. US Department of Health and Human Services Rockville, MD 2006.

[2] U.S. Department of Health and Human Services. The Health Consequences of Smoking—50 Years of Progress: A Report of the Surgeon General,. In: Public Health Service CfDCaP, National Center for Chronic Disease Prevention and Health Promotion, Office on Smoking and Health, editor. US Department of Health and Human Services Rockville, MD 2014.

[3] National Center for Health in Public Housing. Demographic Facts—Residents Living in Public Housing Arlington, VA2015 Available: http://www.namgt.com/hphr/pdfs/demographicfacts.pdf. Accessed 2015 Jun 29.

[4] U.S. Department of Housing and Urban Development. HUD's Public Housing Program Washington, DC2015. Available: http://portal.hud.gov/hudportal/HUD?src=/topics/rental_assistance/phprog. Accessed 2015 Jun 29.

[5] Wilson KM, Klein JD, Blumkin AK, Gottlieb M, Winickoff JP. Tobacco-smoke exposure in children who live in multiunit housing. Pediatrics. 2011;127(1):85–92. Epub 2010/12/15. doi: peds.2010-2046 [pii]. 10.1542/peds.2010-2046 . 21149434

[6] American Society for Heating Refrigerating and Air-Conditioning Engineers. ASHRAE Position Document on Environmental Tobacco Smoke Atlanta, GA2013. Available: http://www.no-smoke.org/pdf/ASHRAE_PD_Environmental_Tobacco_Smoke_2013.pdf. Accessed 2015 Jan 14.

[7] Kraev TA, Adamkiewicz G, Hammond SK, Spengler JD. Indoor concentrations of nicotine in low-income, multi-unit housing: associations with smoking behaviours and housing characteristics. Tob Control. 2009;18(6):438–44. Epub 2009/08/15. doi: tc.2009.029728 [pii]. 10.1136/tc.2009.029728 . 19679890 PMC5624306

[8] Russo ET, Hulse TE, Adamkiewicz G, Levy DE, Bethune L, Kane J, et al Comparison of Indoor Air Quality in Smoke-Permitted and Smoke-Free Multiunit Housing: Findings From the Boston Housing Authority. Nicotine Tob Res. 2014. Epub 2014/08/27. doi: ntu146 [pii]. 10.1093/ntr/ntu146 . 25156526 PMC4837992

[9] Kaufman R, Babb S, O'Halloran A, Asman K, Bishop E, Tynan M, et al Vital signs: nonsmokers' exposure to secondhand smoke—United States, 1999–2008. MMWR Morb Mortal Wkly Rep. 2010;59(35):1141–6. Epub 2010/09/11. doi: mm5935a4 [pii]. . 20829748

[10] Max W, Sung HY, Shi Y. Who is exposed to secondhand smoke? Self-reported and serum cotinine measured exposure in the U.S., 1999–2006. Int J Environ Res Public Health. 2009;6(5):1633–48. Epub 2009/06/23. 10.3390/ijerph6051633 19543411 PMC2697933

[11] Matt GE, Quintana PJ, Zakarian JM, Fortmann AL, Chatfield DA, Hoh E, et al When smokers move out and non-smokers move in: residential thirdhand smoke pollution and exposure. Tob Control. 2011;20(1):e1. Epub 2010/11/03. doi: tc.2010.037382 [pii]. 10.1136/tc.2010.037382 21037269 PMC3666918

[12] Homa DM, Neff LJ, King BA, Caraballo RS, Bunnell RE, Babb SD, et al Vital signs: disparities in nonsmokers' exposure to secondhand smoke—United States, 1999–2012. MMWR Morb Mortal Wkly Rep. 2015;64(4):103–8. Epub 2015/02/06. doi: mm6404a7 [pii]. . 25654612 PMC4584848

[13] US Department of Housing and Urban Development. Non-smoking policies in public housing. 2009. Available: http://www.hud.gov/offices/pih/publications/notices/09/pih2009-21.pdf. Accessed 2015 Jan 14.

[14] Fargen J. Mayor Thomas Menino lights up battle of the butt. The Boston Herald. January 31, 2010 January 31, 2010;Sect. News.

[15] Ferrer B. Executive Director—Boston Public Health Commission, personal communication. In: Levy D, editor. Boston, MA2010.

[16] Benowitz NL. Cotinine as a biomarker of environmental tobacco smoke exposure. Epidemiol Rev. 1996;18(2):188–204. Epub 1996/01/01. . 9021312 10.1093/oxfordjournals.epirev.a017925

[17] Jacob P III, Yu L, Duan M, Ramos L, Yturralde O, Benowitz NL. Determination of the nicotine metabolites cotinine and trans-3'-hydroxycotinine in biologic fluids of smokers and non-smokers using liquid chromatography-tandem mass spectrometry: biomarkers for tobacco smoke exposure and for phenotyping cytochrome P450 2A6 activity. J Chromatogr B Analyt Technol Biomed Life Sci. 2011;879(3–4):267–76. Epub 2011/01/07. doi: S1570-0232(10)00767-1 [pii]. 10.1016/j.jchromb.2010.12.012 21208832 PMC3050598

[18] SRNT Subcommittee on Biochemical Verification. Biochemical verification of tobacco use and cessation. Nicotine Tob Res. 2002;4(2):149–59. Epub 2002/05/25. 10.1080/14622200210123581 . 12028847

[19] Hammond SK, Sorensen G, Youngstrom R, Ockene JK. Occupational exposure to environmental tobacco smoke. JAMA. 1995;274(12):956–60. Epub 1995/09/27. . 7674526

[20] Eisner MD, Katz PP, Yelin EH, Hammond SK, Blanc PD. Measurement of environmental tobacco smoke exposure among adults with asthma. Environ Health Perspect. 2001;109(8):809–14. Epub 2001/09/21. doi: sc271_5_1835 [pii]. 11564616 10.1289/ehp.01109809 PMC1240408

[21] Pion M, Givel MS. Airport smoking rooms don't work. Tob Control. 2004;13 Suppl 1:i37–40. Epub 2004/02/27. 14985615 10.1136/tc.2003.005447 PMC1766150

[22] Miesner EA, Rudnick SN, Hu FC, Spengler JD, Preller L, Ozkaynak H, et al Particulate and nicotine sampling in public facilities and offices. JAPCA. 1989;39(12):1577–82. Epub 1989/12/01. . 2607365 10.1080/08940630.1989.10466652

[23] Hammond SK. Exposure of U.S. workers to environmental tobacco smoke. Environ Health Perspect. 1999;107 Suppl 2:329–40. Epub 1999/06/03. 10350518 10.1289/ehp.99107s2329 PMC1566276

[24] Hammond SK, Leaderer BP. A diffusion monitor to measure exposure to passive smoking. Environ Sci Technol. 1987;21(5):494–7. Epub 1987/05/01. 10.1021/es00159a012 . 22296139

[25] Hammond SK. Collection and analysis of Nicotine as a marker for environmental tobacco smoke. Atmospheric Environment. 1987;21(2):457–62.

[26] Rubin DB. Estimating causal effects from large data sets using propensity scores. Ann Intern Med. 1997;127(8 Pt 2):757–63. Epub 1998/02/12. . 9382394 10.7326/0003-4819-127-8_part_2-199710151-00064

[27] Levy DE, Rigotti NA, Winickoff JP. Tobacco smoke exposure in a sample of Boston public housing residents. Am J Prev Med. 2013;44(1):63–6. Epub 2012/12/21. doi: S0749-3797(12)00715-5 [pii]. 10.1016/j.amepre.2012.09.048 . 23253651

[28] Arku RE, Adamkiewicz G, Vallarino J, Spengler JD, Levy DE. Seasonal variability in environmental tobacco smoke exposure in public housing developments. Indoor Air. 2014 Epub 2014/04/23. 10.1111/ina.12121 24750252 PMC4201978

[29] U.S. Environmental Protection Agency. Blood Cotinine Level 2010. Available: http://cfpub.epa.gov/eroe/index.cfm?fuseaction=detail.viewInd&lv=list.listbyalpha&r=223968&subtop=343. Accessed 2012 Feb 14.

